# Mindful Age and Technology: a Qualitative Analysis of a Tablet/Smartphone App Intervention Designed for Older Adults

**DOI:** 10.1007/s12124-020-09580-x

**Published:** 2020-10-29

**Authors:** Francesco Vailati Riboni, Isabel Sadowski, Benedetta Comazzi, Francesco Pagnini

**Affiliations:** 1 Department of Psychology, Università Cattolica del Sacro Cuore, Milan, Italy; 2 Department of Educational and Counselling Psychology, McGill University, Montreal, Canada; 3 IRCCS Santa Maria Nascente, Fondazione Don Gnocchi, Milan, Italy; 4 Department of Psychology, Harvard University, Cambridge, MA USA

**Keywords:** Older adults, Aging, Technology, Psychological interventions, Clinical psychology

## Abstract

The global population is aging while modern healthcare systems are responding with limited success to the growing care demands of the senior population. Capitalizing on recent technological advancements, new ways to improve older adults’ quality of life have recently been implemented. The current study investigated, from a qualitative point of view, the utility of a mindfulness-based smartphone application for older adults. A description of the older adults’ experience with the smartphone application designed to enhance well-being and mindfulness will be presented. Participants’general beliefs about the benefits of technology for personal well-being will also be discussed. 68 older adults were recruited from different education centers for seniors. Participants were randomly assigned to two groups: a) a treatment group, which received the smartphone application intervention (*n* = 34), or b) a waitlist control group (n = 34). The experimental intervention included the utilization of a smartphone app designed specifically for improving older adult well-being and mindfulness levels. Participants completed semi-structured interviews evaluating participants’ treatment experience and technology-acceptance at recruitment (T0, baseline) and post-intervention (T1, post-intervention). Through thematic analysis, four themes were identified from verbatim responses of both interviews: Utility of technology for health, Impressions of technology, Mindful-benefits of smartphone application usage, and Smartphone application usage as a means to improve interpersonal relationships. Participants showed a positive experience of the app intervention. Qualitative analysis underlined the main Mindfulness-benefits reported by participants and the potentially crucial role of “Langerian” mindfulness in the relationship between older adults and health technology.

## Introduction

The world population is aging while modern healthcare systems are responding with limited success to the growing care demands of the senior population with chronic conditions, creating long-term economic consequences (Kim et al. 2017). The older adult population in Europe and North America is projected to grow from approximately 261.4 million in 2017 to 370 million by 2050, with five of the ten most aged countries or areas in the world expected to be in Europe (Kowal et al. 2012).

This demographic change will require new strategies and solutions for dealing effectively with older adults’ medical issues and psychological well-being (Stewart 2014). Capitalizing on recent technological advancements, new ways to improve older adults’ quality of life have recently been implemented to encourage healthier lifestyles, conduct non-invasive assessments and deliver distance interventions (Cattivelli et al. 2018; Kim et al. 2017; Preschl 2011). New or improved technologies (e.g., e-health, telemedicine, assistive technology, robotics or home care and institution-based healthcare monitoring) have been developed and integrated into older adults’ daily life routines (Bercovitz and Pagnini 2016; Caley and Sidhu 2010; Calvo and Peters 2013; Lattanzio et al. 2014; Wootton 2012).

Technology developed to meet the needs of older adults has been specifically labeled Gerontechnology (Bouma et al. 2009). Gerontechnology may have the capability to improve treatment quality and accessibility while empowering patient-oriented treatment (Kvedar et al. 2014). Evidence from the literature seems to suggest that tech-interventions targeting older adults’ well-being could improve cognitive performances through positive effects on memory, attention, reaction time and global cognitive ability (Ball et al. 2002; Gamberini et al. n.d.; Smith et al. 2009). The integration of advanced technology in psychological and behavioral programs for older adults has been recognized as a promising solution for many issues, including depression, anxiety, and mild cognitive impairment (Vailati Riboni et al. 2020).

Although most of the results discussed in the literature appear encouraging, it must be noted that this could be due to publication bias favoring papers reporting significant or positive outcomes. Cultural and social variables are often underestimated by researchers, although some studies have investigated their role in influencing seniors’ learning capability of specific technology (Bercovitz and Pagnini 2016).

For example, age-related stereotypes held by the senior population may affect the way older adults approach and experience new technologies. In line with Levy’s Stereotype Embodiment Theory (Levy 2009), common stereotypes depicting older adults as not fully capable of dealing with smartphone devices and applications, or unattracted to the tech-field, may be internalized by this specific population.

Seniors’ positive self-perception is another significant factor in the aging process, affecting older adults’ psychophysical health(Levy et al. 2002). Additionally, negative age-based stereotypes may undermine a functional active-aging process (Hehman and Bugental 2013).

However, recent literature suggests that mindfulness practices, described as the simple process of paying attention to novelty with a conscious and non-judgmental attitude, could lead older adults to both positive adaptations of cognitive schemas and improved emotional self-regulation, which play a fundamental role in successful aging (Levy et al. 2015; Rowe and Kahn 1997). Flexibility, openness, curiosity, awareness, and creativity are central factors of a mindful mindset. These variables play a key role in the aging process, from both a psychological and biological perspective (Langer et al. 2010; Bercovitz and Pagnini 2016; Langer et al. 2010).

Mindfulness-based approaches represent potentially useful interventions for increasing seniors’ awareness of aging stereotypes, enabling older adults’ ability to realize when automatic or fixed cognitive schemas have been activated (Langer et al. 2010). The aforementioned barriers preventing older adults from experiencing positive outcomes related to technology integration in their healthcare might be positively addressed through mindfulness practice (Bercovitz and Pagnini 2016).

Given the need for technological solutions to better assist the rapidly increasing aging population, and the potential for mindfulness to improve various quality of life outcomes, the current study investigated the utility of a new mindfulness-based smartphone application for older adults. Using qualitative data compiled from semi-structured interviews, the aims of this study were to:

- describe older adults’ experience with a smartphone application designed to enhance well-being and mindfulness.
- identify participants’ beliefs about the psychological benefits achieved through the mindfulness-based smartphone application.

### A New Mindfulness-App

In the present study, a new Mindfulness-App was developed. The creation of a new tool was carried out by the research team for several reasons:

- The lack of a similar App in the Italian language
- A design process specifically targeting older adult users
- The promotion of mental openness, awareness and cognitive flexibility within one App

**Fig. 1.**
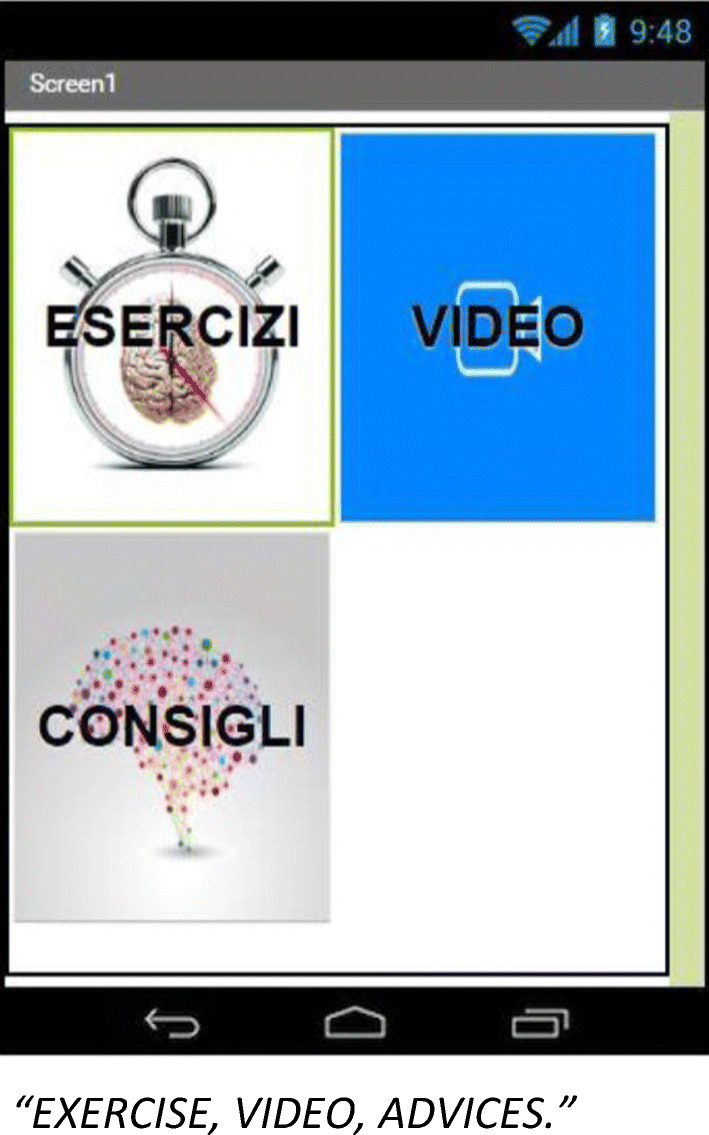
Main menu

The App was developed using MIT App Inventor Software (http://appinventor.mit. edu/explore/), an intuitive, user-friendly, visual programming environment that allows laypeople to build fully functional apps for smartphones and tablets. The blocks-based tool facilitates the creation of complex, high-impact apps in significantly less time than traditional programming environments. Our team carefully developed the App to obtain the most intuitive design, specifically created for older adults. For example, few colors were chosen, with each one identifying a specific button throughout the entire App’s environment (Fig. 1). Large fonts were selected to avoid vision difficulties reading the App’s instructions. The entire app’s design was built accounting for age-related physical and cognitive impairments (Mohadisdudis and Ali 2015). The App’s system was set up to balance information exchange to and from the user. Data provided to the user were presented slowly, while the system’s feedback towards the older adults was faster. The users’ answers were stored and shared with the research team through an automatic App feature. Through the continuous reception of data, it was possible to assess whether participants were actively engaged in the execution of the exercises and therefore in the research program.

**Fig. 2.**
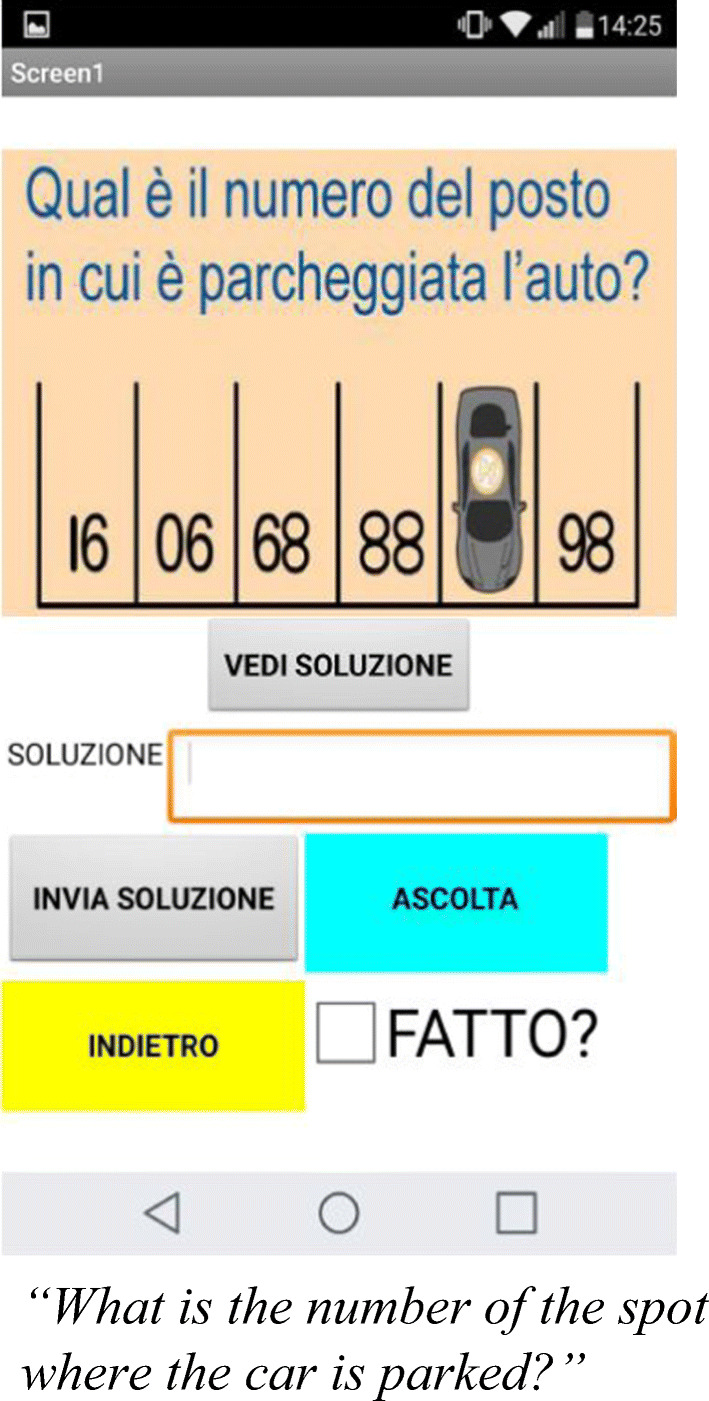
Logical reasoning exercise

**Fig. 3.**
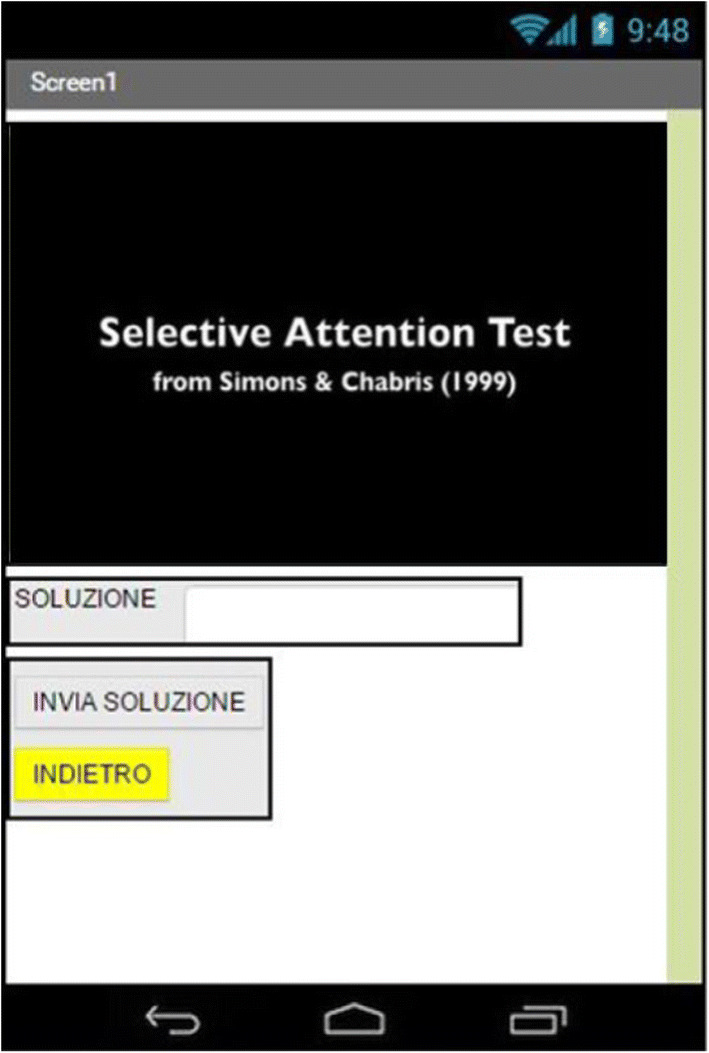
Attention test

Mindfulness-related videos and audio materials were also included in the App to facilitate usability and the learning process. The final product consisted of different mindfulness theoretical explanations, in addition to fourteen exercises connected to logical reasoning and developing mindfulness abilities. The theoretical feature consisted of a) explanatory videos presenting theoretical, scientific and practical principles on which mindfulness practice have been developed, with a particular focus on the possible benefits obtainable through a mindful approach to daily life events; b) pre-recorded audio files giving pieces of information on mindful aging and mindfulness practice; and c) brief texts explaining mindfulness and mindfulness-related general exercises. The first block of seven exercises was specifically designed to empower and stimulate users’decision-making process, as well as awareness. The exercises prompted older adult users to focus on their cognitive process and to reflect on their own choices, in order to promote self-awareness(Figs. 2 and 3).

**Fig. 4.**
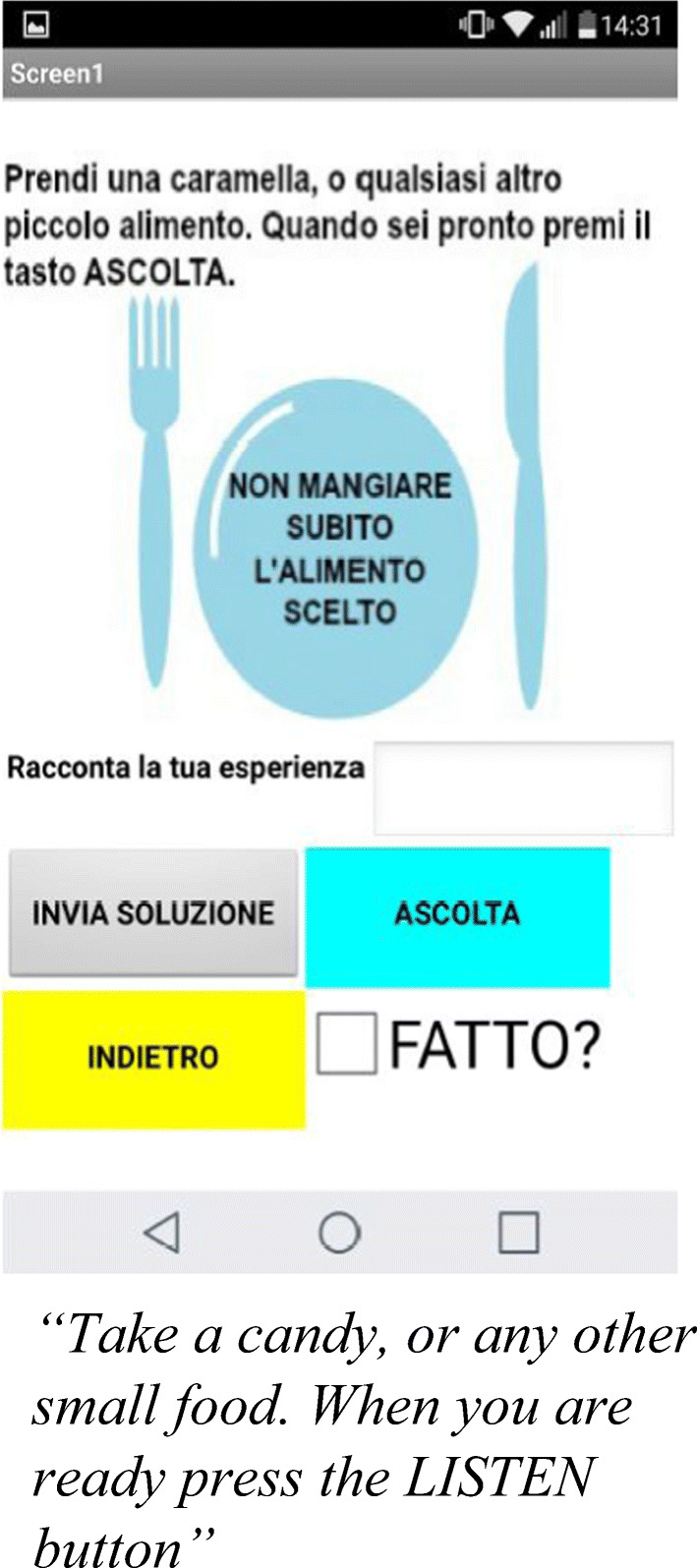
Mindful exercise

The second block of seven exercises focused on the development of lateral thinking, challenging users with creative riddles, logical tests, or behavioral challenges for daily situations (i.e. brushing teeth with the left hand). Within the first seven exercises, users were cognitively stimulated by the different puzzling tasks, ensuring adherence to the study protocol due to the combination of highly ludic engagement experience with accessible positive reinforcements coming from the tasks’ solutions. A different approach was required by the remaining tasks, stimulating creativity, and out of the box mind-sets, with clear references to everyday situations (Fig. 4).

## Method

### Participants and Procedure

Within the context of a larger study (Vailati Riboni et al. 2018), we recruited a sample of 68 older adults, from different education centers for seniors in the city of Milan, Italy.

Inclusion criteria were:

- Age > 65
- Mini-Mental State Examinations (MMSE) > 18. The MMSE is a common psychometric instrument used to assess individual cognitive performance in clinical practice (Folstein et al. 1975).
- Absence of severe neurological or medical conditions, based on medical records.

The final sample was mainly female (71%; *M*_*age*_ = 72, *SD* = 6.00). Regarding educational level, 6% had an elementary degree, 25% had a middle school degree, 53% had a high school degree, and 16% had a master’s degree. At the time of the study, 89% of the sample were employed, and only 11% had retired. Approximately half of the sample (55%) were living with a partner, 32% were living alone, and 13% were living with a relative or a friend. Most of the sample had previous experience with smartphone Apps (80%), and only 20% of the entire sample had no previous experience of any kind with smartphone Apps.

After providing informed consent, participants were randomly assigned to two groups: a) a treatment group, which received the new smartphone application intervention (*n* = 34), and b) a waitlist control group *(n* = 34) which had to wait until the end of the trial before receiving the App.

The experimental intervention included the utilization of the smartphone app designed specifically for improving older adult well-being and mindfulness levels. The entire intervention was designed to be completed in 14 days, with only one exercise per day to be completed.

Participants were able to perform all the exercises on their own, while researchers could monitor participant progress through an automatic feature inserted in the app. Participant attrition did not occur throughout the entire intervention.

### Data Collection

All 34 treatment-group participants completed semi-structured interviews evaluating their treatment experience and acceptance of technology. The first interview was conducted at recruitment (T0, baseline), while the second was conducted after the end of treatment (T1, post-intervention). The semi-structured interviews allowed participants to express themselves freely while enabling the interviewer to follow a logical focus. Participants were interviewed by two researchers (F.V.R. & B.C.). Interviews lasted between 30 min and an hour. All interviews were recorded and transcribed.

### Qualitative Data Analysis

Verbatim responses from both interviews were analyzed using thematic analysis, in line with Braun and Clarke’s (2006) guidelines for approaching qualitative data in psychology (Braun and Clarke 2006). Thematic analysis’s flexibility, as well as its theoretical freedom and commonly shared guidelines for the performance of this particular qualitative method, guided this method selection. The present study used an exploratory approach to the thematic analysis (Braun and Clarke 2006). In line with Braun and Clarke’s (2006) recommendations to increase analysis clarity and reliability, researchers must first describe what they considered to be a theme before actually performing the analysis (Braun and Clarke 2006). In the current study, themes were considered and classified as key factors of the examined experience that showed some kind of pattern across all data. Given the exploratory nature of the current research project, an inductive approach was followed during the process of theme identification. Thematic analysis, however, does not occur in what could be considered an “epistemological vacuum”, and in this particular study researchers’ theoretical background and objectives (i.e. our mindfulness approach) could have potentially affected data analysis (Braun and Clarke 2006; Pouli et al. 2014). Adhering to the adopted methodological procedure, data analysis involved a recursive process between the following phases: a) familiarisation with the data, b) generation of initial codes, c) theme search, d) theme review, e) theme definition, and f) report production (Braun and Clarke 2006).

Participants’ verbatim responses were read and re-read by the researchers (F.V.R., I.S., & B.C) in order to better familiarize themselves with the full material. Initial codes were then produced to systematically identify key features of the data. After all data were coded and collated, different codes were sorted into potential themes. In this phase, the relationship between codes, themes, and subthemes was first examined, and a preliminary sense of the significance of individual themes was developed (Braun and Clarke 2006). Participants’ themes were then refined, taking into consideration individual theme validity regarding the full data set. All themes were reviewed until they described coherent and logical patterns significant to the experimental intervention; marginal themes were dropped. When a satisfactory thematic map of the data was reached, labels were given to each highlighted theme. Transcripts were independently coded by the research team to ensure higher reliability and re-discussed if needed until mutual consensus on the theme classification was achieved.

## Results

Participants’answers from the semi-structured interviews offered interesting descriptions of older adults’ general beliefs regarding the benefits of technology for personal well-being, as well as on their experience with the smartphone App designed to enhance well-being and mindfulness. The following four themes were identified from the data:

1. Utility of technology for health
2. Impressions of technology
3. Mindful benefits of smartphone application usage
4. Smartphone application usage as a means to improve interpersonal relationships

### Is Technology Useful for Health?

Participants were asked if they thought that technology could be useful for health. After analyzing participant responses, three categories were identified: “Yes”, “No”, and “Unsure”.

Participants most frequently indicated that technology was useful for health (category 1; 68%), while 20% of participants indicated that they were unsure of technology’s utility in healthcare (category 3). In contrast, only 12% of participants did not agree that technology was useful for health (category 2).

Accounts of technology’s utility as a health tool predominantly focused on how technology could be useful in elderly populations, for example in the improvement of cognitive functioning and medical interventions.

*Participant 13: Yes, [technology is] useful for keeping your mind exercised and challenged…it’s like going to a gym, where you can do something to get in shape…to keep your mind in good shape and healthy...to improve your situation.*

*Participant 4: Technology could be useful for improving memory and could be very useful [for health] in general.*

Additionally, many participants predicted that technology’s usefulness for health would improve in the future;

*Participant 6: Technology could be useful [for health], but in the near future. For example, in robotic systems for the elderly population.*

Participant responses revealed a general perception that technology for healthcare might be useful in the future, but that they did not currently believe it was aiding their health.

### What Were Participants’ Impressions of Technology?

At baseline, participant impressions of technology could be grouped into five categories, ranging from positive (1) to negative (5): “Technology as useful”, “Technology as an interesting element”, “Technology as something valid, if not over-used”, “Technology as addictive”, and “Technology as scary”.

Most participants thought technology was useful. In particular, they highlighted its value as a beneficial tool to improve daily life;

*Participant 12: Technology is progress, it's an activity, it's indispensable for humanity.*

*Participant 20: [Technology is] really useful for your daily life.*

Other participants thought technology was something interesting, although not completely clear;

*Participant 11: Technology is quite interesting, even if I feel like I am not getting it completely, but it’s fascinating.*

*Participant 29: [Technology is] something that has always interested me. Sometimes*

*I try to understand it more…*

However, despite a generally positive view towards technology, many participants mentioned their perceptions of technology’s potentially addictive qualities, as well as its possibility to be used for negative or frivolous reasons;

*Participant 15: Technology is a good thing unless you become addicted to it.*

*Participant 24: It's useful, but I don't think it's ok to use it for social media… people spend too much time on those things, instead of actually talking to real people… strangers can use social media to trick you, or to try to steal things from you…*

In contrast, some participants were strongly against technology and thought that it was frightening and responsible for stealing human jobs;

*Participant 28: Technology steals works from humans. It is a negative thing.*

*Participant 10: Technology is dangerous. I don’t like it because I can’t control it completely, and it’s scary not to have control over something like that.*

After using the application, four categories were identified regarding experimental group response to whether their impressions of technology had improved:

1. “Yes”,
2. “Yes, but only slightly as their original impression was positive”,
3. “No, because their opinion was already positive”, and
4. “No”.

Participants most frequently stated that their impressions of technology improved after using the app, with 34% in category 1, and 24% in category 2. Approximately one-third of participants (31%) reported no improvement because their opinion was already positive, and 11% did not report any improvement.

Participants were also asked whether they were more curious about technology after using the app for fourteen days. Based on responses, three categories were established:

1. “Yes”,
2. “No, due to prior curiosity about technology use before using the app”, and
3. “No”,

In general, participants were curious about technology; with 46% stating they were more curious after using the application (category 1), and 45% stating that they were curious prior to using the app (category 2). The remaining 9% of the sample (category 3) stated that they continued to be incurious about technology.

### Were Mindful-Benefits Gained from Application Use?

Participants in the experimental group were generally positive about using the application and the majority (86.3%) indicated that they acquired some type of benefit as a result of the mindful components featured within the App. Mindful-benefits of application usage fell into three sub-categories:

1. “Increased flexibility of perspective-taking and lateral thinking”,
2. “Improved cognitive ability”, and
3. “No benefits”.

Participants in category 1 (65%) expressed enthusiasm regarding the ability of the mindful app to change the way they thought about and approached basic tasks of daily life;

*Participant 4: It was a great experience and now I'm able to consider alternative solutions to daily problems. The game tasks were easy to follow, and thanks to them I was willing to try those things I was learning even outside of the App.*

Additionally, they found that using the app helped them to consider things from unconventional or more mindful viewpoints and enabled them to evolve their personal perspectives;

*Participant 7: It helped me to focus on many things from an unconventional point of view.*

*Participant 17: The advantage of using the application was that I gained awareness of the fact that I'm pretty limited in my way of thinking, but now I have a broader point of view.*

Participants in Category 2 (27%) emphasized that one of the main benefits of using the app was that it produced a deeper way of thinking:

*Participant 8: Using your brain more. Thinking about stuff in a deeper way. I have tried to use the mindful information, and they somehow work, they really do.*

*Participant 13: It made me think deeper.*

The Mindfulness features of the Apps seem to have been appreciated by most participants, and despite the short period of use, were potentially able to affect participants’ mind-sets. Benefits highlighted by the interviews seem in line with the general outcomes reported in the relevant literature describing mindfulness integration and acceptability in older adult samples (Bercovitz and Pagnini 2016).

Amongst the remaining 8% who did not find the application to be beneficial, the most commonly cited reasons were that they found the exercises to be too easy and that the application included information they were already familiar with.

### Did Application Usage Improve Participants’ Interpersonal Relationships?

Given that loneliness is a common affliction in the older adult population, this analysis investigated whether application usage improved experimental group participants’ ability to connect with others in their social networks and/or strengthened their pre-existing interpersonal relationships. The mindfulness social component featured within the App was designed to encourage participants’ social interactions and social involvement. Additionally, one of the main theoretical goals of mindfulness practice is to increase feelings of empathy and compassion, resultantly improving individuals’ interpersonal skills (Langer and Moldoveanu 2000). Findings demonstrated that participants’ opinions as to whether the application improved their relationships were quite polarized. For instance, more than half of the participants (56.5%) did not find that their relationships improved as a result of using the app. Amongst these participants, many felt that the application’s focus was much more targeted to personal improvement, and they did not believe it affected their relationships with others. In contrast, those who felt that their relationships meaningfully improved (26.1%) were quite enthusiastic about the application’s ability to help them with their interpersonal interactions;

*Participant 16: Yes, I talked a lot about my experience with other people. I think it takes time to gain trust in new situations, but the application has helped me a lot.*

*Participant 4: Yes, I feel empowered and more open to outside experience.*

As such, it appears that some participants may view this application as a useful tool to build and improve relationships.

## Discussion

The aim of this paper was to describe participants’ experiences with an e-health mindful smartphone application designed to improve psychological well-being via mindfulness and cognitive exercises, through an analysis of their narratives collected via semi-structured interviews. Smartphone applications are already affecting how health can be promoted in our society, especially within the older adult population (Bakker et al. 2016; Kratzke et al. 2012). A considerable number of applications on the market are now dealing with mental health, teaching users new ways to handle stress-related problems, or relaxation techniques deriving from mindful practice. The increasing proliferation of these Apps could indicate a certain degree of effectiveness(Miller 2012). More in-depth research on specific and long-term outcomes is still necessary. In line with recent literature on technology acceptance in older adults, participants perceived technology as something generally positive and useful for their health (Arnaert et al. 2007; Gaggioli and Riva 2013). However, the obtained results suggest that this perception appears to be not entirely positive. Participants’ responses revealed that from their point of view, current technology does not yet fully meet their specific health needs, but that they believe it may succeed at doing so in the near future. Moreover, a few negatives beliefs or fears about technology persisted after exposure to the e-health application, although these were not shared by the majority of the sample. Nonetheless, results from the analysis of the second theme (i.e., participants’ impressions of technology) indicated that participants who used the smartphone application for fourteen days showed greater openness towards technology in general and an increased curiosity towards it. The mindfulness training feature within the smartphone application could have been at least partially responsible for the modification of the perspective of the individual and their openness towards technology. The importance of the mindfulness components included in the treatment was further highlighted in the third theme, (i.e., benefits gained from application use). From the participants’ point of view, the main effects on their psychological health were described as increased flexibility of perspective-taking or lateral thinking, an improved ability to approach basic tasks of daily life, and an increased capacity to consider things from unconventional viewpoints. In line with the Langerian definition of mindfulness, it is possible to support the hypothesis that these results are at least partially attributable to the mindfulness features of the smartphone application, rather than to the cognitive components (Langer and Moldoveanu 2000). Mindfulness has been integrated into recent and innovative research protocols for older adults, showing encouraging results (Pagnini et al. 2019).

With regard to the final theme highlighted by the interviews (i.e., did application usage improve participants’ interpersonal relationships), the experience with this specific e-health application had small effects on participants’ interpersonal relationships. Still, those few individuals who experienced some kind of improvement in their relationships highlighted the application’s ability to help them with their social interaction. It is not clear whether this effect could be directly attributed to any specific features of the app or rather to the experience of the experimentation itself, but this application shows potential as an empowering tool to build and improve relationships for some older individuals.

The sample size could potentially be one limitation of the present study, as well as the short duration of the App’s utilization. Further longitudinal studies may be better able to investigate long- term effects. Most participants had high academic achievement and previous experiences using smartphone applications, potentially creating a bias in the present study.

Results from this study appear to show a positive experience of the App intervention for most participants. This qualitative analysis emphasizes the potential role of Langerian mindfulness when exploring the relationship between older adults and health technology. The mindfulness component of the current smartphone App may improve results in terms of older adults’ openness and acceptance of health technology in their daily living.

Qualitative findings from this study highlight the great potential for positive outcomes in older adults via better integration between technology and psychology. The App appears to be clinically useful at promoting older adults’ psychological well-being. Future studies are needed in order to better understand the generational gap between older adults’ psychological needs, Gerontechnology, and clinical practice.
